# *Hif-2**α*** programs oxygen chemosensitivity in chromaffin cells

**DOI:** 10.1172/JCI174661

**Published:** 2024-08-06

**Authors:** Maria Prange-Barczynska, Holly A. Jones, Yoichiro Sugimoto, Xiaotong Cheng, Joanna D.C.C. Lima, Indrika Ratnayaka, Gillian Douglas, Keith J. Buckler, Peter J. Ratcliffe, Thomas P. Keeley, Tammie Bishop

**Affiliations:** 1 Target Discovery Institute and; 2 Ludwig Institute for Cancer Research, University of Oxford, Oxford, United Kingdom.; 3 The Francis Crick Institute, London, United Kingdom.; 4 Max Delbrück Center for Molecular Medicine in the Helmholtz Association, Berlin, Germany.; 5 DZHK (German Centre for Cardiovascular Research), Partner Site Berlin, Berlin, Germany.; 6 BHF Centre of Research Excellence, Division of Cardiovascular Medicine, Radcliffe Department of Medicine, John Radcliffe Hospital, University of Oxford, Oxford, United Kingdom.; 7 Department of Physiology, Anatomy & Genetics, University of Oxford, Oxford, United Kingdom.

**Keywords:** Development, Oncology, Embryonic development, Hypoxia

## Abstract

The study of transcription factors that determine specialized neuronal functions has provided invaluable insights into the physiology of the nervous system. Peripheral chemoreceptors are neurone-like electrophysiologically excitable cells that link the oxygen concentration of arterial blood to the neuronal control of breathing. In the adult, this oxygen chemosensitivity is exemplified by type I cells of the carotid body, and recent work has revealed one isoform of the hypoxia-inducible transcription factor (HIF), HIF-2α, as having a nonredundant role in the development and function of that organ. Here, we show that activation of HIF-2α, including isolated overexpression of HIF-2α but not HIF-1α, is sufficient to induce oxygen chemosensitivity in adult adrenal medulla. This phenotypic change in the adrenal medulla was associated with retention of extra-adrenal paraganglioma-like tissues resembling the fetal organ of Zuckerkandl, which also manifests oxygen chemosensitivity. Acquisition of chemosensitivity was associated with changes in the adrenal medullary expression of gene classes that are ordinarily characteristic of the carotid body, including G protein regulators and atypical subunits of mitochondrial cytochrome oxidase. Overall, the findings suggest that, at least in certain tissues, HIF-2α acts as a phenotypic driver for cells that display oxygen chemosensitivity, thus linking 2 major oxygen-sensing systems.

## Abstract 2

Induction of electrophysiological oxygen chemosensitivity by the hypoxia inducible transcription factor HIF-2alpha links two major systems of vertebrate oxygen sensing

## Graphical abstract

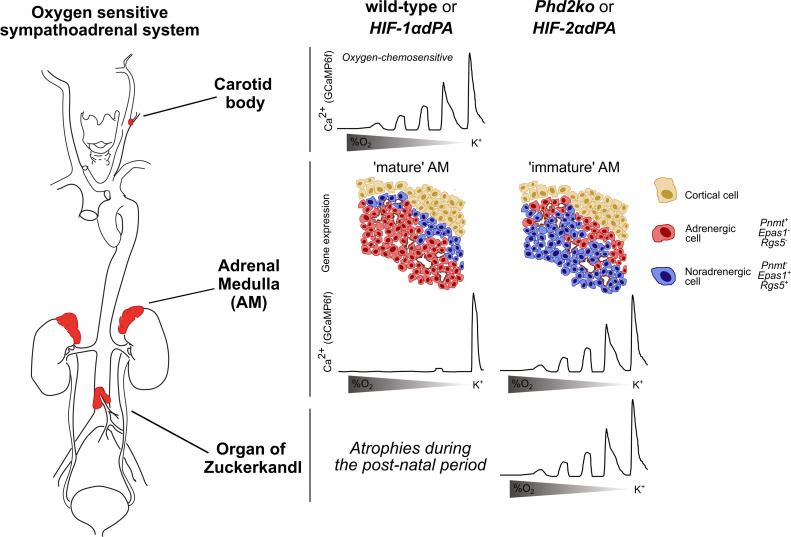

## Introduction

One of the most vital, but still incompletely understood, functions in homeostatic physiology is that of the arterial chemoreceptors that control breathing in response to changes in blood oxygen, carbon dioxide, and other stresses (1–4). This chemosensitivity is generally considered to be a restricted property of specialized cells within the central nervous system and the type I cells of the carotid body (CB), often termed peripheral chemoreceptors (4). CB type I cells respond to low arterial oxygen by K^+^ channel inhibition, resulting in membrane depolarization, voltage-gated Ca^2+^ influx, and dense core vesicle release to excite afferent neurones and stimulate respiration. Nevertheless, despite decades of intense study, the mechanisms that generate and transduce these oxygen-sensitive signals are incompletely understood (1–4).

More recently, a route to the understanding of these processes has been opened by the elucidation of oxygen-sensing mechanisms that transduce transcriptional responses to changes in oxygen availability via hypoxia-inducible transcription factors (HIFs) (2, 5, 6). HIF is an α/β heterodimeric transcription factor whose α subunit is hydroxylated in the presence of oxygen by HIF prolyl hydroxylases (PHDs) to target it for proteasomal degradation via the von Hippel–Lindau (VHL) ubiquitin E3 ligase. In hypoxia, HIF-α subunits escape degradation and dimerize with HIF-β to activate an extensive transcriptional cascade that adapts cells to hypoxia. In keeping with the importance of oxygen homeostasis to animal life, all animals possess at least one HIF-PHD-VHL triad. However, gene duplication events at the base of vertebrate evolution have generated multiple HIF-α and PHD isoforms (7). Thus, mammalian species generally possess 3 PHDs (PHD1, -2, and -3) and 3 HIF-α isoforms (HIF-1α, HIF-2α, and HIF-3α).

Of particular interest is the HIF-2α isoform (otherwise known as endothelial PAS protein 1 and encoded by the *EPAS1* gene), which has been extensively characterized and which shows features consistent with being a more “modern” isoform connected to the function of specialized oxygen-delivery organs, such as lung ventilation and blood vascular systems that are found in higher animals (8–10). To date, one of the most striking of these connections is to the oxygen chemosensitive type I cells of the CB. HIF-2α is expressed at uniquely high levels in type I cells of the CB, and genetic ablation of HIF-2α has revealed essential functions in CB development and in CB oxygen chemosensitivity, including the augmentation of hypoxic ventilatory response after acclimatization to hypoxia (8, 11–17).

In the present work, we sought to determine whether the expression of HIF-2α might be sufficient to induce oxygen chemosensitivity. Here, we show that overexpression of HIF-2α, either by inactivation of the principal PHD (PHD2) or by forced expression of HIF-2α alone, is sufficient to induce marked chemosensitivity in the adult adrenal medulla (AM), which is largely unresponsive in adult life (18–20). The induction of chemosensitivity in the AM was associated with bidirectional changes in the expression of genes that are ordinarily more or less strongly expressed in the CB versus the AM. Alterations in the adult AM were accompanied by the retention of the organ of Zuckerkandl (OZ), which normally atrophies after birth (21), and ectopic adrenal medullary tissue, both of which also manifested oxygen chemosensitivity. Given reports of oxygen chemosensitivity in the fetal adrenal gland (18, 22, 23), these findings are consistent with the maintenance or induction of a fetal-like state in AM and related tissues. The sufficiency of HIF-2α alone to drive these phenotypes suggests a direct impact on the physiology of oxygen chemosensitivity and opens a route to mechanistic understanding.

## Results

### Phd2 inactivation in the AM induces CB-enriched gene expression.

With the aim of better understanding oxygen homeostasis, substantial effort has focused on defining interactions between transcriptional signaling by the HIF pathway and oxygen chemosensitivity in the CB. As part of this work, we observed that inactivation of the principal oxygen sensor that regulates HIF (PHD2) resulted in paraganglioma-like (PGL-like) changes in the CB, AM, and related tissues (12, 24). To pursue this further, we intercrossed mice to obtain *Phd2^fl/fl^* (which we term control or WT) and *Phd2^fl/fl^;ThCre* (*Phd2KO*) animals manifesting tyrosine hydroxylase–restricted (TH-restricted) inactivation of *Phd2* in the CB and AM and performed transcriptomic studies on those tissues. Pooled mRNA from 10 CBs or AMs of animals of each genotype was analyzed using RNA-Seq.

**Figure 1:**
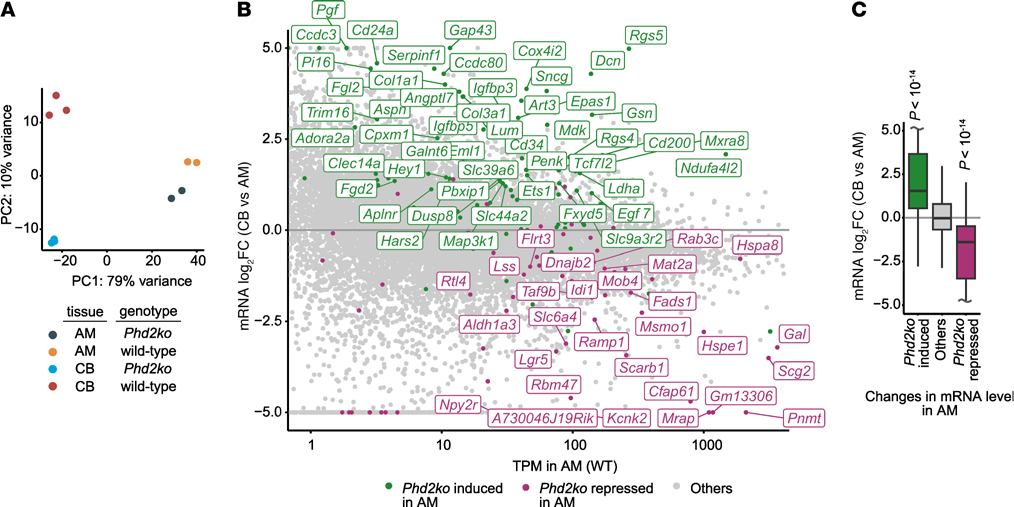
RNA-Seq of *Phd2KO* versus WT AM and CBs from young adult mice. (**A**) Principal component analysis (PCA) of bulk RNA-Seq of CB and AM from *Phd2KO* and WT mice; RNA was extracted from 5 pairs of approximately 2-month-old animals per biological replicate; *n* = 3 or 2 biological replicates for CB or AM, respectively. PC1, first principal component; PC2, second principal component. (**B**) Individual genes that are induced (green) or repressed (purple) by *Phd2KO* in the AM (absolute value of log_2_ fold change > log_2_[1.5] and FDR < 0.1; likelihood ratio test) are overlaid onto genes differentially expressed in WT CB versus WT AM. Gene names are shown for a subset whose mRNA abundance in transcripts per million (TPM) in WT or *Phd2KO* AM is greater than 10. (**C**) Data from **B** shown as a box plot. The fold difference in mRNA abundance in WT CB versus WT AM for genes induced (green) or repressed (purple) by *Phd2KO* in the AM was compared against that for all other genes (gray) using the 2-sided Mann-Whitney *U* test. *n* = 108 (*Phd2KO* induced); *n* = 15,032 (others); *n* = 75 (*Phd2KO* repressed).

Principal component analysis of these data indicated that differences between the CB and AM are the largest source of variance between the datasets, followed by the status of the *Phd2* gene (Figure 1A). Although some established HIF target genes such as *Ldha* and *Bnip3* (25, 26) were upregulated in both the CB and AM upon *Phd2* inactivation, overall changes in mRNA abundance by *Phd2* inactivation showed limited correlation (Spearman’s ρ approximately 0.1) between these tissues (Supplemental Figure 1A; supplemental material available online with this article; https://doi.org/10.1172/JCI174661DS1). Principal component analyses also indicated that *Phd2* inactivation in the AM results in a modest overall shift in gene expression toward that of the CB (Figure 1A). To investigate this further, we focused on the subset of genes that were differentially expressed following *Phd2* inactivation in the AM. Remarkably, our analysis showed that a large majority of the genes induced by *Phd2KO* in the AM were more highly expressed in the CB compared with those in the AM, whereas a large majority of those repressed by *Phd2KO* in the AM were expressed at lower levels in the CB (Figure 1, B and C). Since the same dataset for WT AM was used in both comparisons (*Phd2KO* versus WT AM, and CB versus AM from WT mice), we went on to determine whether this relationship is observed when using an independently generated comparator dataset comparing the CB versus AM (27). The results were concordant (Supplemental Figure 1B versus Figure 1C), indicating the robustness of the finding. Overall, transcriptomic analyses demonstrated that *Phd2KO* in the AM alters the expression of a subset of its transcripts toward a pattern that is similar to that in the CB. Notably, these include a number of genes involved in mitochondrial energy metabolism (*Cox4i2*, *Ndufa4l2*, and *Higd1c*), G protein signaling (*Rgs5* and *Adora2a*), and *Epas1* itself — all genes that are, or are implicated to be, involved in oxygen chemosensitivity (13, 16, 28–30) (see Supplemental Table 1 for full transcriptomic dataset; Supplemental Table 2 for genes that are upregulated by *Phd2* inactivation in both the CB and the AM; Supplemental Table 3 for genes that are CB enriched and upregulated by *Phd2* inactivation in the AM; Supplemental Table 4 for data on genes that are, or have been implicated to be, involved in oxygen chemosensitivity). Of note, *Phd2KO* in the AM (or CB) did not induce expression of the CB-enriched HIF-2 target gene Olfr78, which is reported to play a role in oxygen chemosensitivity (27, 29, 31).

### Phd2 inactivation confers oxygen chemosensitivity on chromaffin cells.

In adult mammals, the oxygen chemosensitivity associated with the CB is normally considered to be tightly restricted to the type I cells in that organ. Other cells, including pulmonary vascular smooth muscle cells and neuroepithelial bodies, also manifest responses to hypoxia that occur on a similarly rapid time scale and may share some mechanisms (1, 32, 33). While the adult AM responds to neurogenic stimuli, the majority of its chromaffin cells are not intrinsically responsive to hypoxia (18–20). However, the above alterations in gene expression raised the interesting possibility that this paradigm might be broken by *Phd2* inactivation and confer CB-like chemosensitivity onto the AM. Since hypoxia is established to promote Ca^2+^ influx in CB type I cells following membrane depolarization (34), changes in intracellular Ca^2+^ concentration were recorded in response to superfusion of tissues with hypoxic solutions (equilibrated to 10%–0% oxygen) or with a solution containing 45 mM K^+^ to maximally depolarize cells. Intracellular Ca^2+^ concentration ([Ca^2+^]_i_) fluctuations were detected by imaging tissues from transgenic mice expressing a genetically encoded calcium indicator: GCaMP6f recombined into the Rosa26 locus and preceded by a restricting “*lox-stop-lox”* sequence (known as *Ai95^fl/+^*). These mice were intercrossed with *ThCre* animals to generate *Ai95^fl/+^;ThCre* mice in which GCaMP6f expression is restricted to TH^+^ cells, including those in the CB and AM. We first confirmed that oxygen chemosensitivity is observed in CBs from *Ai95^fl/+^;ThCre* mice. In these experiments, superfused whole CBs were exposed to graded hypoxia by switching between superfusate equilibrated to 10%, 5%, 1%, or 0% oxygen or a sham control in which a switch was made between perfusates with identical oxygenation. In these and all other experiments, superfusion with high (45 mM) K^+^ was included in the experimental protocol to define the regions that might respond to hypoxia and provide normalization for other interventions. Hypoxia resulted in a rapid (within seconds) and reversible increase in [Ca^2+^]_i_ (Supplemental Figure 2). Further, CB type I cells responded to graded hypoxic stimuli with graded rises in [Ca^2+^]_i_, with responses to anoxia reaching approximately 80% of the maximum increase in [Ca^2+^]_i_ achieved with high K^+^.

**Figure 2:**
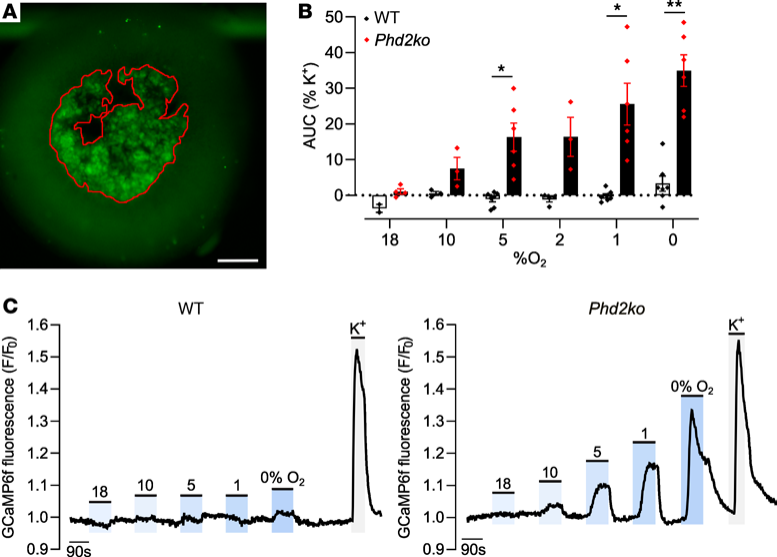
Ca^2+^ imaging showing oxygen chemosensitivity in *Phd2KO* (*Phd2^fl/fl^;Ai95^fl/+^;ThCre*), but not WT (*Ai95^fl/+^;ThCre*), AM from young adult mice. (**A**) Representative image showing GFP fluorescence in an AM slice from a mouse expressing genetically encoded calcium indicator (GCaMP6f), the expression of which is restricted to TH^+^ cells (*Ai95^fl/+^;ThCre*, referred to as WT in Figures 5, 6, and 8) and perfused with 45 mM K^+^. Red outline shows an example of the K^+^-responsive region of interest from which fluorescence is quantified. Scale bar: 0.2 mm. (**B**) Average AUC corresponding to each hypoxic (or sham, 18% O_2_) stimulus in **C**. Figures are normalized as percentages of AUC under 45 mM K^+^ signal. Data are represented as mean ± SEM with individual data points overlaid in this and subsequent figures. Data were analyzed by a mixed-effects 2-way ANOVA, with 18% O_2_ excluded from analysis: variation due to change in oxygen tension, *P* < 0.0001; variation due to *Phd2* inactivation, *P* = 0.0004, followed by Šidák’s multiple-comparisons test on pairwise comparisons at each oxygen level. *P < 0.05; **P < 0.01. (**C**) Representative traces showing fluorescence (F) in the AM (averaged across the K^+^-responsive region as per red outline in image **A**), background corrected, and normalized to the fluorescence at the beginning of the recording (F_0_) to give F/F_0_; shaded areas highlight the time for which the indicated stimuli are applied: hypoxia (18%–0% O_2_) or 45 mM K^+^ (in this and all subsequent figures depicting GCaMP6f recordings).

Next, we sought to test sensitivity to hypoxia in the AM. To obtain a more consistent representation of the tissue (which is larger than, and has a different anatomy from, the CB, being encircled by the adrenal cortex), adrenal gland slices rather than whole tissues were used. Exposure to high K^+^ to induce membrane depolarization resulted in a large increase in [Ca^2+^]_i_, which was used to define the region of interest for potential oxygen chemosensitivity (see Figure 2A). Unlike the CB, AM from *Ai95^fl/+^;ThCre* mice that are WT for *Phd2* (referred to as WT hereafter) were almost completely unresponsive to hypoxia or anoxia (Figure 2, B and C). Since it has been reported that neonatal AM cells are chemosensitive and that this property is reduced but not completely lost postnatally (18–20), we assessed whether any responsiveness to hypoxia could be observed by closer examination of AM from *Ai95^fl/+^;ThCre* mice at a higher magnification; this identified small regions with minor oxygen chemosensitivity (Supplemental Figure 3). In striking contrast, AM from *Phd2^fl/fl^;Ai95^fl/+^;ThCre* animals (referred to as *Phd2KO* hereafter) manifested extensive and robust oxygen chemosensitivity when tested under identical conditions compared with AM from WT mice (Figure 2, B and C). The increase in [Ca^2+^]_i_ in response to hypoxia was rapid (within seconds) as well as reversible. Furthermore, the response was graded in response to the severity of the hypoxic challenge, as observed in the CB, with stimulation by anoxia reaching approximately 35% of the maximum response obtained with high K^+^ (Figure 2B). These experiments therefore reveal that TH-restricted inactivation of *Phd2* is associated with the upregulation of a number of genes in the AM whose expression is high in the CB and with the acquisition of oxygen-sensitive excitability, as manifested by rapid and reversible rises in [Ca^2+^]_i_ in response to hypoxia.

### Phd2 inactivation results in the formation of oxygen chemosensitive abdominal PGLs.

**Figure 3:**
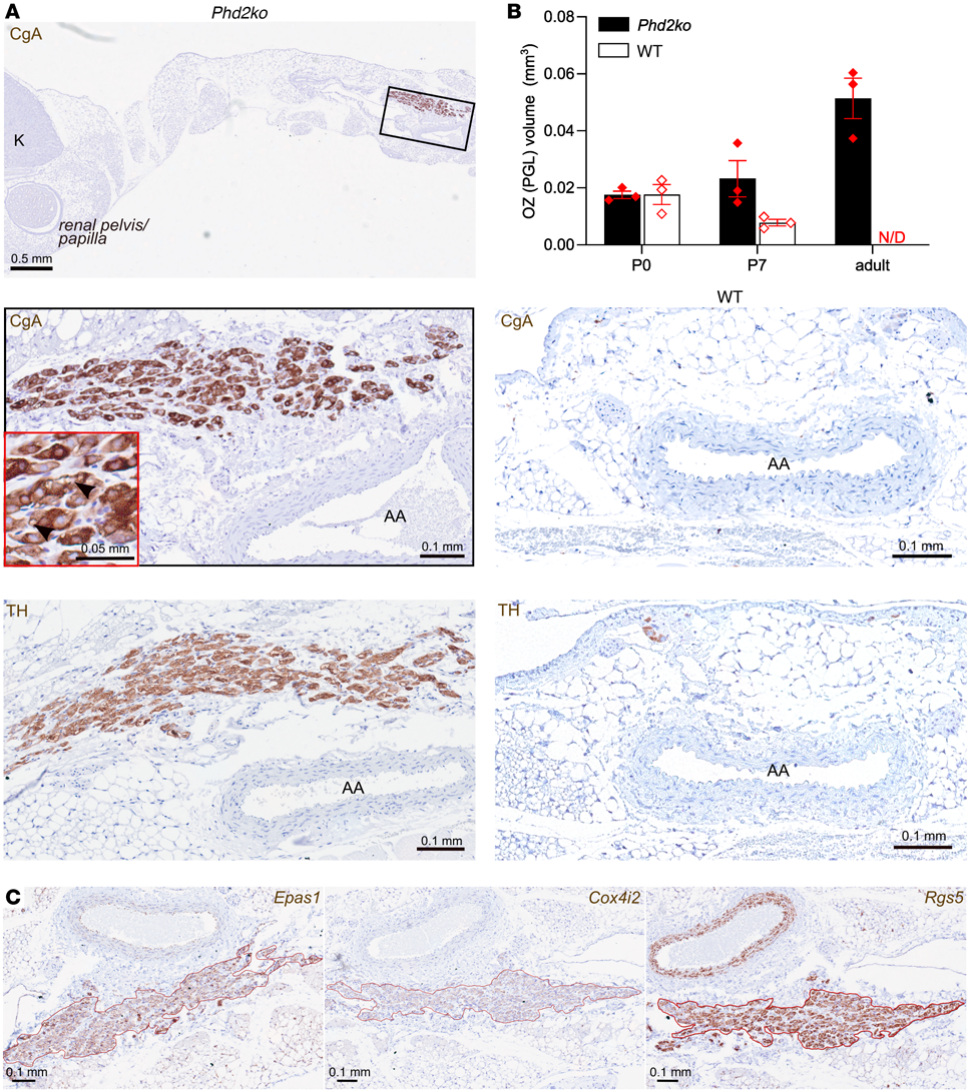
Abdominal OZ PGL in *Phd2KO*, but not WT, young adult mice. (**A**) CgA (top left and middle 2 panels) and TH (bottom panels) immunohistochemistry in transverse sections of abdominal aorta (AA) and kidneys (K) in *Phd2KO* (left) and WT (right) adult mice. Low magnification image shows a transverse section of abdominal cavity, with the aorta-adjacent OZ PGL shown at higher magnification (see red insert for detailed cellular morphology showing “clearing” within some CgA^+^ cells within the PGL). This structure is absent in a comparable region of the WT mouse. (**B**) OZ or OZ PGL volume based on CgA^+^ structure in abdominal cavities in neonatal (P0 and P7) and adult *Phd2KO* and WT mice. No OZ PGL-like structure was detected in adult WT mice (N/D). (**C**) In situ hybridization for *Epas1*, *Cox4i2*, and *Rgs5* mRNA in the OZ PGL from an adult *Phd2KO* mouse.

In a previous study, we noted that *Phd2KO* results in the presence of collections of ectopic TH^+^ chromaffin cells, both within and surrounding the adrenal cortex (24). In light of the above findings, we sought to investigate this further. To that end, a systematic examination of the abdominal region was performed by transversely sectioning the area encompassing the superior to inferior mesenteric arteries and immunostaining for the presence of ectopic TH^+^ cells. Since the abdominal region contains several TH^+^ sympathetic ganglia, immunostaining for chromogranin A (CgA) was also performed to identify chromaffin cells. This revealed a large TH^+^ and CgA^+^ structure adjacent to the abdominal aorta and close to the inferior mesenteric artery (Figure 3A). This structure was absent in WT mice, and its position and CgA positivity were strongly suggestive of a retained OZ. The OZ is a fetal structure that acts as the main source of catecholamines during development, but disappears postnatally in WT mice (21, 35, 36). Our findings reveal that *Phd2KO* results in the apparent retention of an OZ-like CgA^+^ and TH^+^ structure in adult mice that was substantially enlarged compared with the OZ in WT, *Phd2KO* fetal, or *Phd2KO* newborn mice (Figure 3B and Supplemental Figure 4). Many of the TH^+^ and CgA^+^ cells had a “cleared” appearance, previously described in PGL-like CBs from these mice (12) as well as OZ PGLs in humans (37, 38). To pursue this resemblance to the CB, we tested the expression of selected genes that were upregulated in *Phd2KO* AM, highly expressed in the CB and/or directly implicated in oxygen chemosensitivity (see above): *Epas1*, encoding HIF-2α; *Rgs5*, encoding a regulator of G protein signaling; and *Cox4i2*, encoding an alternative regulatory subunit of mitochondrial cytochrome *c* oxidase (Figure 1B). All these genes were strongly expressed in this abdominal OZ PGL-like structure (Figure 3C).

**Figure 4:**
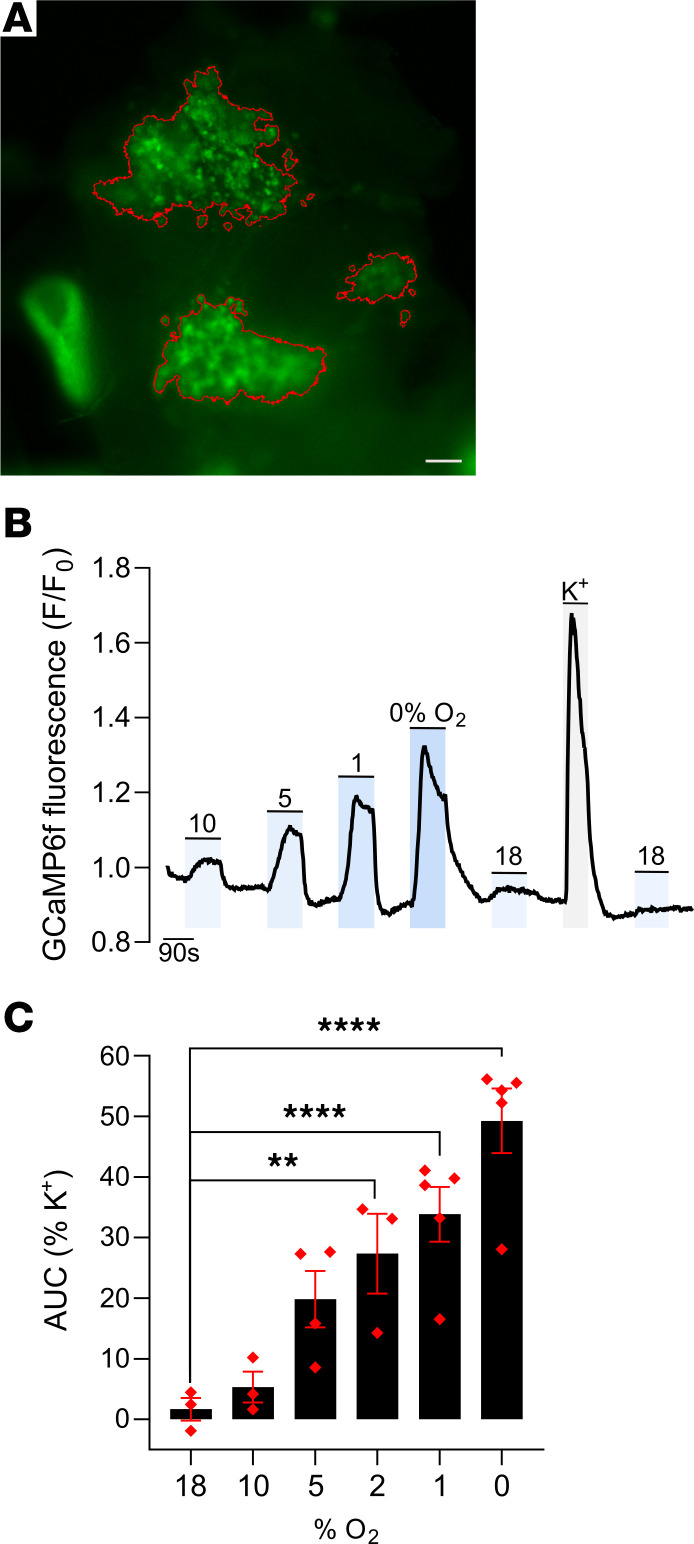
Oxygen chemosensitivity in the abdominal OZ PGL of a *Phd2KO* young adult mouse. (**A**) Representative image showing GFP fluorescence in an OZ PGL from an adult *Phd2KO* mouse perfused with 45 mM K^+^. Red outlines show the K^+^ responsive regions of interest, from which GCaMP6f fluorescence is quantified in **B** and **C**. Scale bar: 0.2 mm. Large bright-green structure visible in the bottom left corner is an adjacent blood vessel. (**B**) Representative trace showing F/F_0_ fluorescence in the K^+^ responsive regions as the tissue is exposed to the indicated stimuli: 10%–0% O_2_, 45 mM K^+^, or sham (18% O_2_). (**C**) Average AUC normalized to the signal in response to 45 mM K^+^ in *Phd2KO* OZ PGLs. Data were analyzed by 1-way, repeated-measures ANOVA: variation due to oxygen tension, *P* = 0.0035, followed by Dunnett’s multiple-comparisons test comparing each stimulus to 18% O_2_. **P < 0.01; ****P < 0.0001.

We therefore sought to determine whether the structure manifests oxygen chemosensitivity. Abdominal OZ PGLs were isolated from GCaMP6f-expressing *Phd2KO* mice and studied by perfusion of whole, subdissected OZ PGL preparations with hypoxic buffers. These experiments revealed a pattern of oxygen chemosensitivity similar to that observed in AM from mice of the same genotype (Figure 4, A–C). Signals were again graded in accordance with oxygen tension, with stimulation by anoxia reaching a proportion of the response to high K^+^ (~50%) similar to that observed in the AM (Figure 4C).

### Doxapram and CO_2_ sensitivity of Phd2KO AMs and abdominal OZ PGLs.

**Figure 5:**
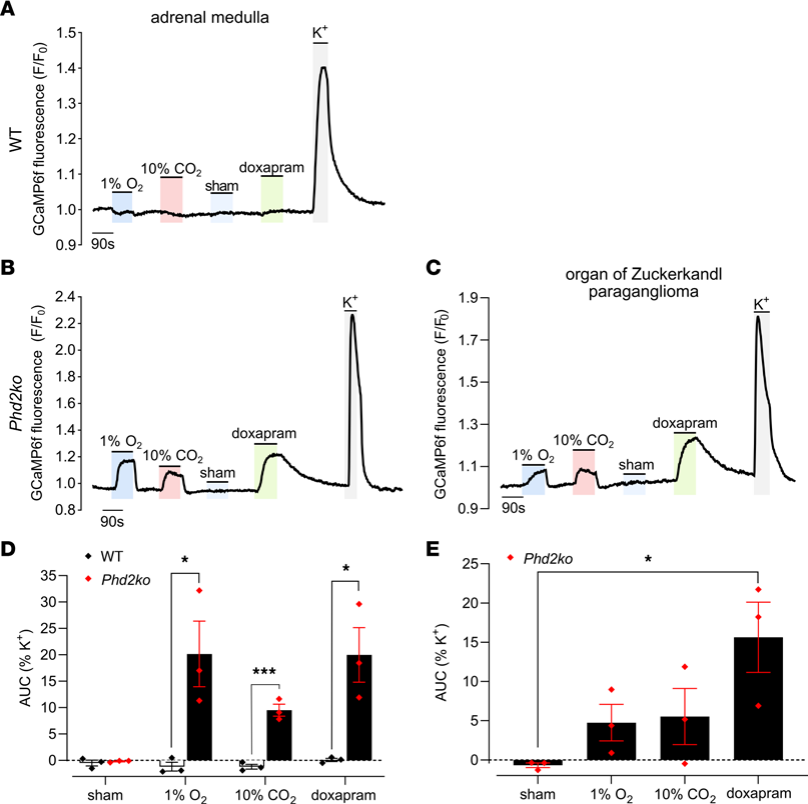
*Phd2* inactivation induces Ca^2+^ mobilization in chromaffin cells in response to 10% CO_2_ and doxapram. Representative traces of GCaMP6f F/F_0_ fluorescence in the K^+^ responsive region of (**A**) WT (*Ai95^fl/+^;ThCre*) and (**B**) *Phd2KO* (*Phd2^fl/fl^;Ai95^fl/+^;ThCre*) AM and (**C**) *Phd2KO* OZ PGL from young adult mice, as the tissues are exposed to indicated stimuli: 1% O_2_, 10% CO_2_, sham test solution (18% O_2_), 50 μM doxapram or 45 mM K^+^. (**D** and **E**) Average AUC normalized as percentage of AUC with 45 mM K^+^ in (**D**) WT and *Phd2KO* AM slices and (**E**) OZ PGL. Data were analyzed by (**D**) 2-tailed, unpaired Student’s *t* tests and (**E**) 1-way ANOVA: variation due to gas stimulus applied, *P* = 0.0334, followed by Dunnett’s multiple-comparisons test against the sham. *P < 0.05; ***P < 0.001.

Next, we sought to further characterize the chemosensitivity of these AM and OZ PGL-like structures in *Phd2KO* mice, in particular their resemblance to CB chemosensitivity. We first tested responses to doxapram (1-ethyl-4-(2-morpholin-4-ylethyl)-3,3-diphenylpyrrolidin-2-one), a central respiratory stimulant whose mode of action is thought to be via direct stimulation of CB type I cells (39). AM were superfused with 50 μM doxapram, which is reported to inhibit Twik-related acid-sensitive K^+^ (TASK) channel activity and evoke an increase in cytosolic Ca^2+^ in dissociated CB type I cells (39). Second, we tested responses to acid/CO_2_, which is also known to excite the CB (40–42). AMs (and to a lesser extent the OZ PGLs) from *Phd2KO*, but not WT, mice were sensitive to both 50 μM doxapram and 10% CO_2_ (Figure 5). Interestingly, neonatal AMs, like the CB, have been reported as being chemosensitive to hypoxia and CO_2_, with these properties being lost during maturation (18–20, 43, 44). Thus, *Phd2* inactivation in the AM appears to result in the retention of neonatal sensitivities to hypoxia and CO_2_ that resemble those observed in the CB.

### Activation of HIF-2α, but not HIF-1α, is sufficient to induce oxygen chemosensitivity.

Inactivation of the principal oxygen sensor PHD2 leads to stabilization of HIF-α subunits and activation of HIF. Several pieces of data have implicated one isoform, HIF-2α, as being necessary for CB development and physiological function and for the PGL-like phenotypes associated with inactivation of *Phd2* (11–13, 16, 17, 24), although some studies have emphasized the importance of HIF-1α (45, 46). Together with the above observations on induction of oxygen chemosensitivity, these findings led us to consider whether overexpression of HIF-2α, or HIF-1α, alone might be sufficient to induce oxygen chemosensitivity in the AM. To address this, a gain-of-function *HIF-2α* allele (*HIF-2αdPA*) or, for comparison, a gain-of-function *HIF-1α* allele (*HIF-1αdPA*), in which the 2 sites of prolyl hydroxylation in HIF-1 or -2α are substituted with alanine residues to prevent hydroxylation and hence convey resistance to degradation by the VHL ubiquitin/proteasomal pathway, was used (47). Activation of these transgenes in *HIF-1* or -*2αdPA^fl/+^;ThCre* mice (referred to as *HIF-1αdPA* or *HIF-2αdPA* hereafter) leads to HIF-1 or -2α overexpression in TH^+^ cells of the CB and AM. This was confirmed by immunoblotting for the hemagglutinin (HA) tag present on HIF-1 or -2αdPA (with somewhat reduced expression of HIF-1α compared with HIF-2α, as has been previously reported; ref. 47) as well as by immunohistochemistry for HIF-2α in the AM, CB, and OZ where present (Supplemental Figures 5 and 6). To demonstrate the activity of this transgene, we first tested for effects of HIF-1 or -2α overexpression on the CB. Consistent with earlier work (48), these experiments demonstrated that (while CBs were ~1.4-fold enlarged in *HIF-1αdPA* mice in comparison with those from WT animals) *HIF-2αdPA* mice developed grossly enlarged (~5 fold) PGL-like CBs (Supplemental Figure 7, A and B). In line with this, we also observed an increase in ventilatory sensitivity to hypoxia in *HIF-2αdPA*, but not *HIF-1αdPA*, mice (Supplemental Figure 7C).

**Figure 6:**
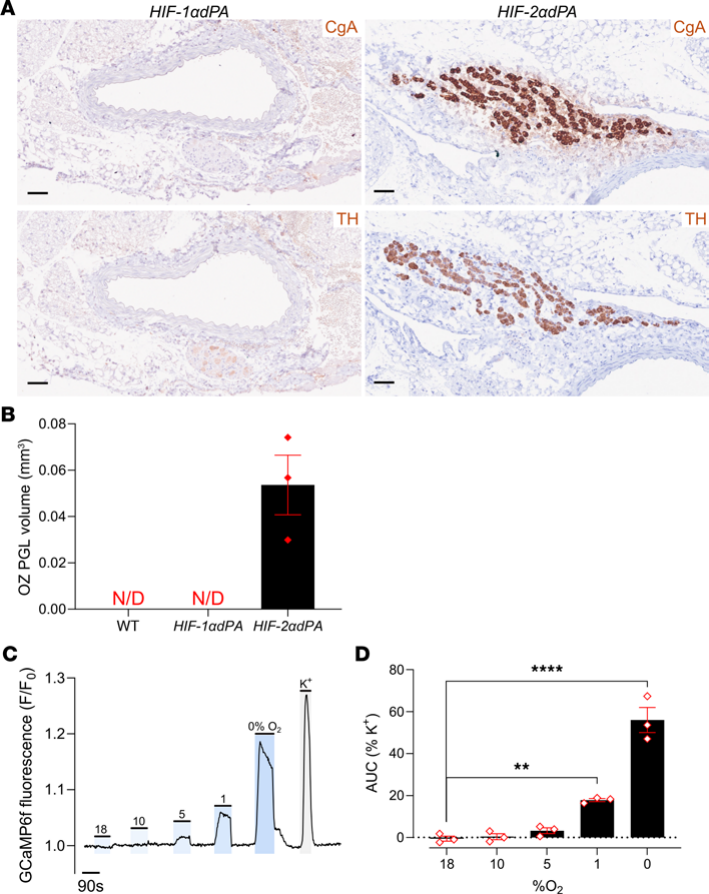
Abdominal OZ PGL in *HIF-2*α*dPA* (*HIF-2*α*dPA^fl/+^;ThCre*) young adult mice. (**A**) CgA (top panels) and TH (bottom panels) immunohistochemistry in OZ PGL shown in transverse sections of abdominal aorta in adult *HIF-2*α*dPA* mice; this structure was not detected in adult *HIF-1*α*dPA* (or WT) mice. Scale bars: 0.05 mm. (**B**) OZ PGL volume based on CgA^+^ structures in abdominal cavities in adult *HIF-2*α*dPA* mice. No OZ PGL-like structure was detected in adult *HIF-1*α*dPA* or WT mice (N/D). (**C**) Representative trace showing F/F_0_ fluorescence in the K^+^ responsive regions in an OZ PGL from an adult *HIF-2*α*dPA* mouse, as the tissue is exposed to indicated stimuli: hypoxia (10%–0% O_2_), 45 mM K^+^ or sham (18% O_2_). (**D**) Average AUC normalized as percentage of AUC under 45 mM K^+^ in *HIF-2*α*dPA* OZ PGLs (with the sham control used for 18% O_2_). Data were analyzed by 1-way ANOVA: variation due to oxygen tension, *P* < 0.0001, followed by Dunnett’s multiple-comparisons test against 18% O_2_. **P < 0.01; ****P *<* 0.0001.

**Figure 7:**
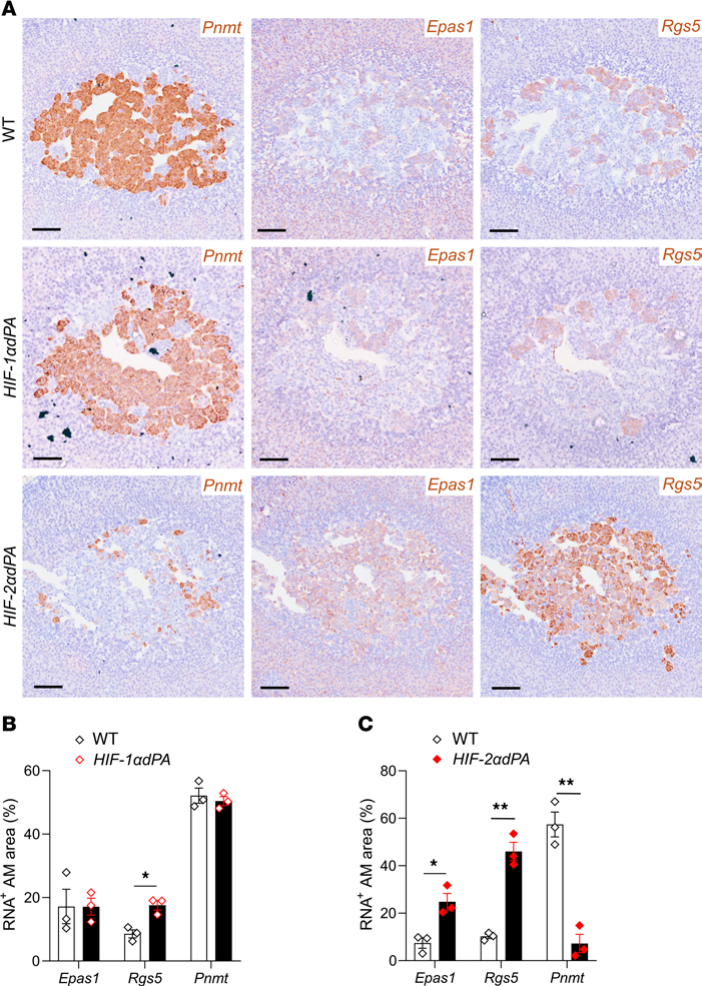
Gene expression in WT, *HIF-1*α*dPA* (*HIF-1*α*dPA^fl/+^;ThCre*), and *HIF-2*α*dPA* (*HIF-2*α*dPA^fl/+^;ThCre*) AM from young adult mice. (**A**) In situ hybridization for *Pnmt*, *Epas1* (*Hif-2*α), and *Rgs5* (brown) in adjacent sections from WT versus *HIF-1*α*dPA* or *HIF-2*α*dPA* mice. Harris hematoxylin counterstain (purple). Scale bars: 0.1 mm. For each mRNA, mean area of expression was compared between WT and (**B**) *HIF-1*α*dPA* or (**C**) *HIF-2*α*dPA* using unpaired Student’s *t* tests with multiple-comparisons *P* value adjustment using the Holm-Šidák’s method. *P < 0.05; **P < 0.01.

**Figure 8:**
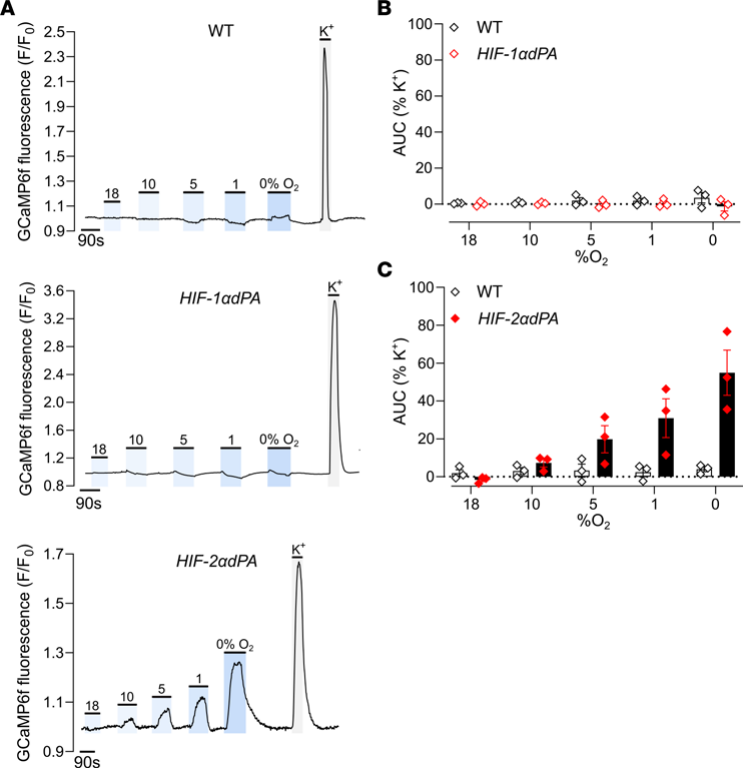
Oxygen chemosensitivity in WT, *HIF-1*α*dPA* (*HIF-1*α*dPA^fl/+^;Ai95^fl/+^;ThCre*), and *HIF-2*α*dPA* (*HIF-2*α*dPA^fl/+^;Ai95^fl/+^;ThCre*) AM from young adult mice. (**A**) Representative traces showing F/F_0_ fluorescence in WT, *HIF-1*α*dPA*, or *HIF-2*α*dPA* AM as the tissue is exposed to the indicated stimuli: 10%–0% O_2_, 45 mM K^+^, or sham (18% O_2_). (**B** and **C**) Average AUC to each hypoxic (or sham, 18% O_2_) stimulus (as recorded in **A**) normalized as percentage of AUC with 45 mM K^+^ in WT versus (**B**) *HIF-1*α*dPA* or (**C**) *HIF-2*α*dPA* mice. Data in **B** and **C** were analyzed by 2-way ANOVA with 18% O_2_ excluded from analysis: variation due to change in oxygen tension, *P* = 0.8029 (**B**), *P* = 0.0054 (**C**); variation due to HIF-1α activation, *P* = 0.1738 (**B**); variation due to HIF-2α activation, *P* = 0.0352 (**C**), followed by a Holm-Šidák’s multiple-comparisons test on pairwise comparisons between WT and *HIF-1/2*α*dPA* at each oxygen level (shown in graphs), which revealed no significant comparisons.

Importantly, anatomical and histological examination of the adrenal glands, periadrenal, and abdominal regions of the *HIF-2αdPA* animals also revealed abnormalities that were essentially identical to those observed in the *Phd2KO* mice; these were entirely absent in *HIF-1αdPA* mice. Particularly striking was the retention of OZ PGL-like tissues in adult *HIF-2αdPA*, but not *HIF-1αdPA*, mice (Figure 6, A and B). As with *Phd2KO* animals, this tissue expressed increased levels of *Rgs5* and *Cox4i2* as well as *Epas1* mRNA (Supplemental Figure 8). In the AM, a switch in the cellular pattern of gene expression toward apparently immature *Pnmt^–^Rgs5^+^Epas1^+^* (where *Pnmt* is defined as phenylethanolamine *N*-methyltransferase) cells was observed in HIF-2α–overexpressing cells (Figure 7, A and C), as is also observed in *Phd2KO* animals (where it is reversed by inactivation of *Hif-2*α, but not *Hif-1*α, ref. 24). This population switch toward immature *Pnmt^–^* noradrenergic cells was not observed with HIF-1α overexpression; however, there was a small significant increase in *Rgs5^+^* (Figure 7B). Robust induction of oxygen chemosensitivity closely similar to that observed in *Phd2KO* mice was observed both in the AM and the abdominal OZ PGLs of *HIF-2αdPA* (*HIF-2αdPA^fl/+^;Ai95^fl/+^;ThCre*), but not *HIF-1αdPA* (*HIF-1αdPA^fl/+^;Ai95^fl/+^;ThCre*), animals (Figure 6, C and D, and Figure 8). Thus, stabilized HIF-2α, but not HIF-1α, is sufficient to drive a PGL-like oxygen-chemosensitive phenotype in chromaffin cells which, under the conditions of these experiments, was essentially identical to that observed with *Phd2* inactivation.

## Discussion

Our findings establish that activation of hypoxia-signaling pathways, either by inactivation of the regulatory PHD2 or by overexpression of one of its targets, HIF-2α, is sufficient to induce oxygen chemosensitivity in the adult AM and that this is specific to that HIF isoform. We observed that induction of oxygen chemosensitivity was associated with a bidirectional shift in expression of selected genes within the AM that coincides with differences in gene expression between the normal CB and the normal AM.

Interestingly, this pattern of gene expression included genes with proposed functions in generating or tuning hypoxic signals from the mitochondria. Thus, expression of *Cox4i2*, an alternative isoform of *Cox4*, which is a nuclear-encoded regulatory subunit of the cytochrome *c* oxidase complex, and *Ndufa4l2*, another nuclear-encoded component of this complex, were both increased in the oxygen-chemosensitive AM of *Phd2KO* mice (Supplemental Tables 1–4). Our findings therefore strengthen the hypothesis that these genes, and their actions on the kinetic properties of cytochrome *c* oxidase, are important determinants of oxygen chemosensitivity (16). Another intriguing finding was the striking upregulation of *Rgs5* transcripts, which encode a regulator of G protein signaling that is a target of the recently recognized oxygen sensitive *N*-terminal cysteine dioxygenase 2-aminoethanethiol dioxygenase (ADO) (49).

Activation of HIF-2α in the AM also results in a marked reduction in *Pnmt* expression and switch to a noradrenaline-secreting phenotype (ref. 24 and this study). Spatial analyses using in situ mRNA hybridization have suggested that this might represent a largely binary switch between 2 populations of cells: *Pnmt^–^* cells secreting noradrenaline and expressing high levels of *Hif-2α*, *Rgs5*, and *Cox4i2* mRNAs and *Pnmt^+^* cells secreting adrenaline and expressing much lower levels of these transcripts. Single-cell transcriptomic analyses have also demonstrated an association of *Hif-2α*, *Rgs5*, and *Cox4i2* mRNAs with lack of expression of *Pnmt* in embryonic and postnatal AM (50). These findings raise an interesting question as to whether oxygen chemosensitivity and a noradrenaline-secreting phenotype are mechanistically linked or simply associated properties. However, this association makes it difficult to draw precise conclusions as to which genes are involved directly in chemosensitivity as opposed to an associated noradrenaline-secreting phenotype. Genes that are differentially expressed in WT CBs (which are chemosensitive but do not have a noradrenergic-secreting phenotype) versus WT AM should assist in this; these genes include those identified above. It is also possible that HIF-2 targets promoting chemosensitivity would be upregulated by HIF-2α in both AM and CB. We have provided these lists as a resource in Supplemental Tables 1–3. Defining their exact mechanistic roles will require further study.

Particularly significant, however, was the sufficiency of forced expression of HIF-2α alone (but not HIF-1α) to create oxygen chemosensitivity. *HIF-2α* mRNA is expressed at strikingly high levels in the CB (8, 12, 14), and it is necessary for the development and oxygen chemosensitivity of that organ and for the enhanced hypoxic ventilatory responses mediated by the CB in hypoxic acclimatization (11–13, 16, 17). Taken together with these observations, the current findings suggest that, among its other functions, HIF-2α appears to be integral to the specification of oxygen chemosensitivity in sympathoadrenal or chromaffin cells. Further, although HIF-1 and HIF-2 bind to an identical core motif and share many target genes (51), our work suggests that oxygen chemosensitivity is mediated by genes that are uniquely regulated by HIF-2, which may include the atypical mitochondrial regulators *Cox4i2*, *Ndufa4l2*, and *Higd1c* or the regulator of G protein signaling *Rgs5* (29).

Interestingly, the induced chemosensitivity in *Phd2KO* and *Hif-2α*-overexpressing AM and that of the retained OZ-like structures was not limited to oxygen chemosensitivity, but included responsiveness to high CO_2_ and doxapram. Since equilibration of the bicarbonate buffer with high CO_2_ will reduce pH, we were not able to distinguish whether the direct sensitivity was to acid or CO_2_ itself. Nevertheless, both high acid/CO_2_ and doxapram are established stimulants of the type I chemosensitive cells in the CB, where the targets are considered to be the TASK channels TASK1 and TASK3 (39, 52). However, TASK1 (*Kcnk3*) and TASK3 (*Kcnk9*) transcripts were neither overexpressed in the CB versus AM nor induced in *Phd2KO* AM (Supplemental Tables 3 and 4), and in line with this, it has been previously reported that HIF-2α does not affect TASK1 or TASK3 expression in the CB (16).

Several pieces of information suggest that the alterations we have observed following intervention on the PHD2/HIF-2 pathway reflect reversion to a fetal-like state. First, the alteration in gene expression involves loss of adrenal medullary expression of *Pnmt*, which has been considered to be a marker of chromaffin cell differentiation (53). Second, the changes involve retention of OZ-like structures that normally characterize the fetal state. Third, the physiological characteristics, chemosensitivity to oxygen and CO_2_, reflect those that have been reported for fetal adrenal tissues (18, 19).

Though further work will be required to understand the exact basis of this phenotypic switch, the current findings have biochemical, physiological, and clinical implications. By recording the induction of oxygen chemosensitivity in a previously largely unresponsive tissue, the work enhances the power of association studies between gene expression and phenotype. More generally, the work provides a model for the study of oxygen chemosensitivity, including future studies as to which of the downstream targets of HIF-2α are required for oxygen chemosensitivity. It also has potential implications for the phenotype of “pseudohypoxic” pheochromocytoma, which typically manifests upregulation of genes in the HIF pathway and is associated with mutations in *HIF-2α*, *PHD2*, *PHD1*, *VHL*, and specific subunits of tricarboxylic acid cycle enzymes succinate dehydrogenase *SDHx*. These mutations are frequently germline or occur in the early postzygotic period and so potentially have developmental consequences (54, 55). It will now be important to determine whether oxygen chemosensitivity is a characteristic feature of these tumors, as awareness of factors stimulating catecholamine release from these tumors is critical for safe clinical management. Further, given the association between hypoxic activation of chemosensing and cell proliferation in the CB (56), the work also raises an interesting question as to whether oxygen chemosensitivity contributes to the growth and development of these tumors.

In summary, our work reveals a link between 2 key systems of oxygen sensing. It should be important in directing future work on the pathogenesis of PGL/pheochromocytoma development and more generally in investigating the fundamental physiological challenge of maintaining oxygen homeostasis.

## Methods

### Sex as a biological variable.

Both female and male mice were used in the experiments presented. Control animals were always sex matched. Sex was not considered as a biological variable.

### Animals.

Experiments were performed on approximately 2-month-old, young adult mice of the relevant genotype and sex-matched controls. Mice were kept in individually ventilated cages with free access to water and food. *Phd2^fl/fl^* and *ThCre* alleles are as previously described (57, 58); *Ai95^fl/+^*, *HIF-1αdPA^fl/+^*, and *HIF-2αdPA^fl/+^* were obtained from Jackson Laboratories (strains 028865, 009673 and 009674) and are as previously described (47, 59). Each mouse line was backcrossed with C57BL/6 for at least 5 generations.

### Immunohistochemistry.

Tissue processing and immunohistochemistry procedures were as described previously (12, 13, 60). Animals were culled with overdose of isoflurane or cervical dislocation and tissues dissected and placed in 4% paraformaldehyde (w/v in PBS) and left to fix at room temperature overnight, then transferred into 70% ethanol and stored at 4°C until processing. Tissues were processed by gradual dehydration in 70%, 90%, and 100% ethanol, then Histo-Clear II (National Diagnostics), and finally wax at 60°C in an automated tissue processor (Excelsior AS, Thermo Scientific), then embedded in paraffin and sectioned with a HM 355S Microtome (Thermo Scientific) to 4 μm thickness. To detect catecholaminergic cells, paraffin sections were immunostained with anti-CgA antibody (1:500 ab68271, Abcam) or anti-TH antibody (1:5000, NB300-109, Novus Biologicals) using the Envision+ Kit (Dako) according to the manufacturer’s instructions and counterstained with modified Harris Hematoxylin (Epredia) differentiated with 1% acetic acid (Merck) for optimal stain intensity. Stained tissues were scanned with the NanoZoomer S210 slide scanner (Hamamatsu) and OZ volume estimated by calculating CgA^+^ area using NDP.View2 software (Hamamatsu) as previously described (12).

### In situ hybridization.

RNA was detected in formalin-fixed, paraffin-embedded tissues (as prepared above) using the RNAScope Manual Assay (ACDBio) per the manufacturer’s instructions, with the following gene-specific probes: *Epas1* (catalog 314371), *Rgs5* (catalog 430181), and *Cox4i2* (catalog 497901). Harris Hematoxylin (Epredia), differentiated with 1% acetic acid for optimal intensity, was used as a counterstain.

### RNA extraction and RNA-Seq.

CBs and AMs were pooled from 5 animals per genotype (i.e., 10 in total for each tissue) to obtain sufficient material, and RNA was extracted using the RNEasy Micro Plus Kit (QIAGEN) according to the manufacturer’s instructions and as described previously (15). Tissues were subdissected into RNAProtect Tissue Reagent (QIAGEN) and placed on ice for 2–5 hours until all tissues were collected. One volume of PBS in diethyl pyrocarbonate–treated H_2_O was then added to Tissue Protect, and tissues were centrifuged at 16,100*g* for 1 minute to collect at the bottom of the tube, allowing removal of the Tissue Protect and PBS mix. Subsequently, 300 μl of ice-cold RLT Plus Buffer (QIAGEN) with 20 μl of 2 M DTT per 1 ml was added and tissues homogenized using the Pro200 Homogenizer (PRO Scientific) at 5 × 6 second pulses for CBs and 4 × 5 second pulses for AMs. Lysate was then centrifuged for 3 minutes at 16,100*g*, 4°C, and supernatant transferred into a clean, RNAse-free tube then immediately frozen on dry ice. Lysates were stored at –80°C for 1–7 days before continuing with RNA extraction. Lysates were thawed on ice and the remaining steps from the QIAGEN kit were carried out at room temperature. RNA was eluted with 14 μl RNAse-free H_2_O and quality assessed using the High Sensitivity RNA ScreenTape (Agilent) per the manufacturer’s instructions. The remaining RNA was used immediately or stored at –80°C. Ultra-low library preparation and RNA-Seq using the NovaSeq 6000 platform were performed by the Genomics Facility at the Wellcome Centre for Human Genetics, University of Oxford.

### RNA-Seq data processing and reference data.

Based on the RNA-Seq data, the transcript abundance was calculated using Salmon (version 1.9.0) (61) with the reference transcript data by GENCODE (version 31) (62). K^+^ channel–related genes were defined as those associated with the Gene Ontology (GO) term GO:0005267 (K^+^ channel activity).

### RNA-Seq data analysis: principal component analysis.

The transcript count data of genes were transformed using the vst function of DESeq2 without considering the sample information, and principal component analysis was performed using the plotPCA function of DESeq2 (63).

### RNA-Seq data analysis: identification of differentially expressed genes.

The fold change and statistical significance of differential gene expression between 2 tissues or upon *Phd2*KO were calculated using DESeq2. For the analysis of the statistical significance of differential gene expression upon *Phd2KO*, the difference between batches of samples was considered using the likelihood ratio test whenever possible. Genes with a FDR of less than 0.1 and an absolute value of log_2_ fold change of greater than log_2_(1.5) were defined as differentially expressed. Genes with a very low expression level were identified by DESeq2, and these genes were excluded from the analyses.

### Carotid bifurcation, OZ, and adrenal gland preparation for live Ca^2+^ imaging.

Mice were culled by terminal anesthesia (or cervical dislocation if CBs not used), and carotid bifurcations, aortas, and adrenal glands were collected into ice-cold PBS. CBs and OZ PGLs were then subdissected in ice-cold PBS and placed on a 100 μm nylon mesh (Warner Instruments) in F12 media (Gibco; Thermo Fisher Scientific) with FBS (Gibco; Thermo Fisher Scientific), penicillin/streptomycin, and l-glutamine, then kept at 37°C, 5% CO_2_ until imaging (for up to 4 hours). Adrenal glands were embedded in 5% low-melting-point agarose (BP1360, Fisher Scientific) in modified Krebs-Henseleit buffer (25 mM NaHCO_3_, 117 mM NaCl, 1 mM NaH_2_PO_4_, 11 mM d-glucose, 4.5 mM KCl, 1 mM MgCl_2_, 25 mM HEPES, pH 7.4) at 39°C and allowed to cool on ice for approximately 5 minutes; 150 μm slices were obtained with a VT100S Vibratome (Leica) using Feather FA-10 carbon blades (Labtech) vibrating at 80 Hz, 0.2 mm/s. Slices were placed in DMEM media containing 10% FBS and 2 mM l-glutamine (Gibco, Thermo Fisher Scientific), then kept at 37°C, 5% CO_2_ for at least 1 hour before imaging (for up to 4 hours).

### Fluorescence imaging.

Tissues were imaged in a custom-built gravity-based perfusion system, with a wide-field Leica DMi8 microscope (Leica Microsystems CMS), configured with a pE340^fura^ light source (CoolLED), a GFP filter cube (excitation filter 470 ± 20 nm, 495 nm dichroic mirror, emission filter 525 ± 25 nm), and a monochrome Prime 95B camera (Photometrics). Krebs-Henseleit buffer–based (25 mM NaHCO_3_, 117 mM NaCl, 1 mM NaH_2_PO_4_, 11 mM d-glucose, 4.5 mM KCl, 1 mM MgCl_2_) test solutions were bubbled with gases using 3-channel Gas Blender 100 Series mixers (100 series, MCQ Instruments) for at least 2 hours prior to the start of the experiment. Solutions were heated in-line upstream of the tissue chamber to achieve a temperature of approximately 37°C in the chamber. Tissues were placed in the perfusion chamber and immobilized with a slice anchor (SHD-22L/15 or SHD-22L/10; Warner Instruments), and control solution was supplied until the start of the experiment. Images were obtained every 2 seconds with a ×40 oil-immersive objective (HC PL FLUOTAR 40×/1.30 OIL 340 nm, Leica Microsystems CMS) used for CBs and a ×10 objective (N PLAN 10×/0.25 achromatic corrected objective, Leica Microsystems CMS) used for OZ and AM. Bright-field images of the AM were taken prior to the start of recording to select the AM area for analysis. Baseline fluorescence was measured for at least 60 seconds prior to the application of the first test. Test solutions were applied for 90 seconds (or up to 60 seconds for K^+^), with at least 120 seconds rest in control solution between tests. For gaseous composition of test solutions, see Supplemental Table 5. Raw data were analyzed using Fiji software (NIH). Average GFP fluorescence intensity across the region of interest was quantified across the *Z*-stack and plotted against time. For background correction, small regions on-tissue but away from K^+^-responsive cells (i.e., cells in which exposure to high K^+^ led to voltage-gated Ca^2+^ influx) were selected.

Additional information on methods used in experiments presented in the Supplemental Figures are provided in the Supplemental Methods.

### Statistics.

All statistical analyses were carried out using Graphpad Prism 9.0 or R (4.2.1) software. Details of statistical tests are included in the figure legends. All data are represented as means ± SEM.

### Study approval.

The animal studies were performed under the Home Office animal work project license (PPL P38BE32DE and PP9828946) and approved by the University of Oxford Medical Science Division Ethical Review Committee, in compliance with the UK Home Office Animals (Scientific Procedures) Act 1986.

### Data availability.

The scripts and pipeline used for the analysis of RNA-Seq are available on GitHub (https://github.com/YoichiroSugimoto/20230620_Phd2KO_in_AM_and_CB, branch name: main, commit ID: 587a1425f403a35a0f0d56c000e2d7477f5de57d). Sequence data generated from this study are available from ArrayExpress (E-MTAB-13163). Values for all data points in graphs are reported in the Supporting Data Values file.

## Author contributions

Experiments were designed by MPB, HAJ, YS, KJB, PJR, TPK, and TB. MPB, HAJ, YS, XC, JDCCL, IR, GD, KJB, PJR, TPK, and TB were involved in data collection and analysis. The manuscript was prepared by MPB, HAJ, YS, PJR, TPK, and TB and reviewed by all authors. Figures were prepared and statistical analyses performed by MPB, HAJ, and YS with input from other authors. PJR, TPK, and TB conceived the study and managed the project. MPB, HAJ, and YS are co–first authors; PJR, TPK and TB are co–senior authors. The order of the co–first authors’ names was determined on the basis of their relative contributions to the study.

## Supplementary Material

Supplemental data

Unedited blot and gel images

Supplemental table 1

Supplemental table 2

Supplemental table 3

Supplemental table 4

Supporting data values

## Version 1 08/06/2024

In-Press Preview

## Version 2 09/17/2024

Electronic publication
