# Supplementary material for: Hif-2α programs oxygen chemosensitivity in chromaffin cells
Source: J Clin Invest. 2024 Aug 6;134(18):e174661. doi: 10.1172/JCI174661 (PMC11405041; doi:10.1172/JCI174661)
Supplement: Supplemental data [file jci-134-174661-s031.pdf]

Hif-2α programmes oxygen chemosensitivity in chromaffin cells

Supplementary materials

Maria Prange-Barczynska[1,2], Holly A. Jones[1,2], Yoichiro Sugimoto[3,4,5], Xiaotong Cheng[1,2], Joanna D. C. C. Lima[1,2], Indrika Ratnayaka[2], Gillian Douglas[6], Keith J. Buckler[7], Peter J. Ratcliffe[1,2,3], Thomas P. Keeley[1,2,7] and Tammie Bishop[1,2,7]

[1]Target Discovery Institute, University of Oxford, Oxford, UK

[2]Ludwig Institute for Cancer Research, University of Oxford, Oxford, UK

[3]The Francis Crick Institute, London, UK

[4]Max Delbrück Center for Molecular Medicine, Berlin, Germany

[5]DZHK (German Centre for Cardiovascular Research), Partner Site Berlin, Berlin, Germany

[6]BHF Centre of Research Excellence, Division of Cardiovascular Medicine, Radcliffe Department of Medicine, John Radcliffe Hospital, University of Oxford, Oxford, UK

[7]Department of Physiology, Anatomy & Genetics, Parks Road, Oxford, UK

Authorship note: MPB, HAJ and YS are joint first authors. PJR, TPK and TB are co-senior authors.

Correspondence to: Peter J. Ratcliffe, Thomas P. Keeley and Tammie Bishop, Target Discovery Institute, Roosevelt Drive, Oxford, OX3 7FZ, UK, Tel: +44-1865-612-680.

peter.ratcliffe@ndm.ox.ac.uk; thomas.keeley@ndm.ox.ac.uk;

tammie.bishop@ndm.ox.ac.uk

Supplementary methods

Experiments presented in Supplementary materials were carried out according to the same protocols as described in the main manuscript, except for the following:

Analysis of cellular heterogeneity in wild-type adrenal medullae

To assess whether a minority population of chromaffin cells within the adult adrenal medulla retained oxygen chemosensitivity, adrenal gland slices from wild-type mice were exposed to graded hypoxia (as described elsewhere) and imaged under higher magnification (HC PL FLUOTAR 40x/1.30 OIL 340 nm, Leica Microsystems CMS). To identify regions of interest (ROI) equivalent to single cells, the resulting images were subjected to a 30 pixel rolling average background selection and all resulting above-threshold ROI selected for quantification. The fluorescence intensity (F/F_0) for each ROI was plotted and all ROI unresponsive to the application of high K^+ were excluded. The remaining ROI were manually sorted according to whether clear electrophysiological responses were observed upon exposure to ≥1%O_2, anoxia or lack of O_2-sensitive response all together (labelled ‘unresponsive’).

HA immunoblotting

Tissues were dissected, lysed in sodium dodecyl sulfate (SDS) lysis buffer (50mM Tris pH 6.8, 2% SDS, 10% glycerol) and sonicated to homogeneity. Samples were normalised for protein concentration using a BCA assay (Pierce, Thermo Fisher Scientific), then mixed with Laemmli sample buffer. Proteins were separated by SDS-polyacrylamide gel electrophoresis then transferred onto a polyvinylidene difluoride membrane (Immobilon-P, Millipore). This was blocked in 4% fat free milk (w/v in phosphate buffered saline, PBS, containing 0.1% Tween20) then incubated overnight with HRP-conjugated primary antibody against HA (3F10, Sigma Aldrich) diluted 1:500 in 4% milk. Chemiluminescence substrate (West Dura, 34076, Thermo Fisher Scientific) was added and bound antibody was detected using a ChemiDoc XRS+ imaging system (BioRad). β-actin (ab49900, Abcam) was used as a loading control.

Multi-layer indirect labelling for HIF-2α

For the detection of HIF-2α, a new signal amplification system was developed in the laboratory. 4 µm formaldehyde-fixed paraffin-embedded sections were deparaffinised, rehydrated and subjected to heat-induced epitope retrieval with the Dako Target Retrieval Solution pH 6.0 (S2369, Agilent) for 30 min at 120°C. Sections were blocked for non-specific protein, avidin and biotin binding and were incubated with anti-HIF-2α primary antibody (1)(PM8; 1:3000) overnight at 4°C. Indirect labelling was performed by serial incubations with secondary HRP-conjugated anti-rabbit antibody (Dako K4003, Agilent; 30 min room temperature, RT), tertiary biotin-conjugated anti-HRP antibody (Rockland 200-4638-0100; 1:250; 2 h RT) and quaternary HRP-conjugated Streptavidin (BD Pharmingen 551321; 1 h RT). Slides were developed using 3,3'-diaminobenzidine chromogen and counterstained with haematoxylin.

Plethysmography

Hypoxic ventilatory response (HVR) was assessed by whole-body plethysmography in awake animals (2, 3): mice were placed in Buxco whole-body plethysmographs (PLY4211, 600 ml, DSI) and allowed to acclimatise to the new environment for 30 min with plethysmographs unsealed and filled with room air. 1.5 $L \cdot min^{-1}$ medical air flow was initiated ~3 min prior to the start of the recording and minute ventilation was measured for 5 min in air, followed by 5 min at 10% O_2/3% CO_2/balance N_2 (supplied from a pressured gas cylinder, BOC) and another 5 min at medical air. Data were normalised to animal's body weight recorded immediately prior to placing the animal in the plethysmograph and plotted against time. HVR was quantified as the difference between the average minute ventilation during the first minute of stable hypoxia (excluding the first 30s after switching the gas supply) and the average minute ventilation during the last minute of normoxia.

References

1. Wiesener MS, et al. Widespread hypoxia-inducible expression of HIF-2alpha in distinct cell populations of different organs. *FASEB J.* 2003;17(2):271-3.
2. Bishop T, et al. Carotid body hyperplasia and enhanced ventilatory responses to hypoxia in mice with heterozygous deficiency of PHD2. *J Physiol.* 2013;591(14):3565-77.
3. Cheng X, et al. Marked and rapid effects of pharmacological HIF-2alpha antagonism on hypoxic ventilatory control. *J Clin Invest.* 2020;130(5):2237-51.
4. Chang AJ, et al. Oxygen regulation of breathing through an olfactory receptor activated by lactate. *Nature.* 2015;527(7577):240-4.

Supplementary Tables

Supplementary Table 1. RNA Sequencing of *Phd2ko* (*Phd2$^{f/f}$;ThCre*) versus wild-type (*Phd2$^{f/f}$*) adrenal medullae (AM) and carotid bodies (CB). RNA expression in *Phd2ko* and wild-type CB and AM are provided for all annotated genes detected in a separate Excel resource.

Supplementary Table 2. Genes that are *Phd2ko* induced in both the carotid body (CB) and adrenal medulla (AM). A subset of Supplementary Table 1, which shows genes that are upregulated by *Phd2ko* in both the AM and CB.

Supplementary Table 3. Genes that are carotid body (CB) enriched and *Phd2ko* induced in the adrenal medulla (AM). A subset of Supplementary Table 1, which shows genes that are enriched in the CB and upregulated by *Phd2ko* in AM.

Supplementary Table 4. Selection of genes from Supplementary Table 1 that are, or have been implicated to be, involved in oxygen chemosensitivity. Provided in a separate Excel resource.

Supplementary Table 5. Gas composition in test solutions used for tissue superfusion in fluorescent imaging experiments. For details of the fluorescence imaging protocol, see the main manuscript. 50 µM doxapram solution was prepared by diluting a 1000x doxapram hydrochloride (USP) stock in DMSO in control solution. Anoxic solution was supplemented with 4 mg·ml^{-1} sodium sulphite (Merck).

Test solution	Compressed air	N_2	CO_2
Control,<br>50 µM doxapram,<br>45 mM K^+	95%	0%	5%
10% O_2	48%	47%	5%
5% O_2	23%	72%	5%
2% O_2	9%	86%	5%
1% O_2<br>Anoxia	2%	93%	5%
10% CO_2	90%	0%	10%

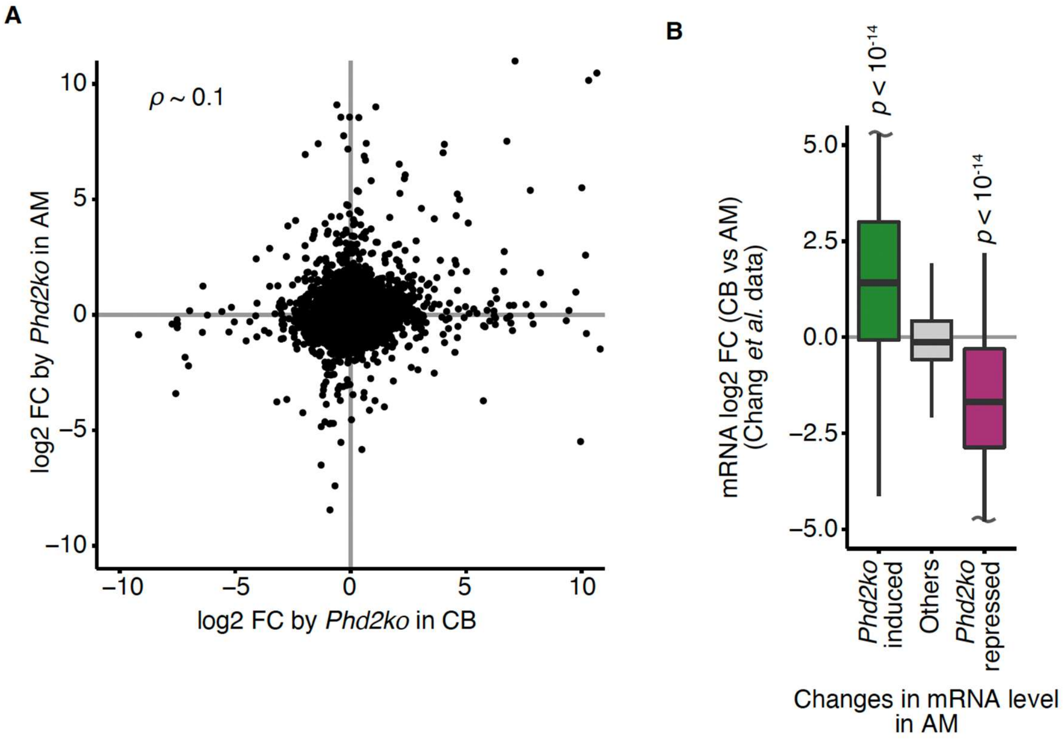

Supplementary Figure 1. RNA Sequencing of *Phd2ko* (*Phd2*$^{f/f}$*;ThCre*) versus wild-type (*Phd2*$^{f/f}$) adrenal medullae (AM) and carotid bodies (CB) from young adult mice. (**A**) Comparison of the effects of *Phd2ko* on mRNA abundance in the carotid body (CB) with that in the adrenal medulla (AM). The correlation was analysed with the Spearman's rank coefficient (n = 9,867). Genes with too little expression (transcript per million ≤ 10 in all of the conditions analysed) were excluded from the analysis. (**B**) Similar to Figure 1C (main text), but the fold difference of mRNA abundance between the CB and AM was calculated using the data from (4) instead of data generated in this paper. The distribution for genes induced (green) or repressed (purple) by *Phd2ko* in the AM was compared against that for all other genes (grey) using the two-sided Mann-Whitney U test (n = 114, 80, and 15,315, respectively).

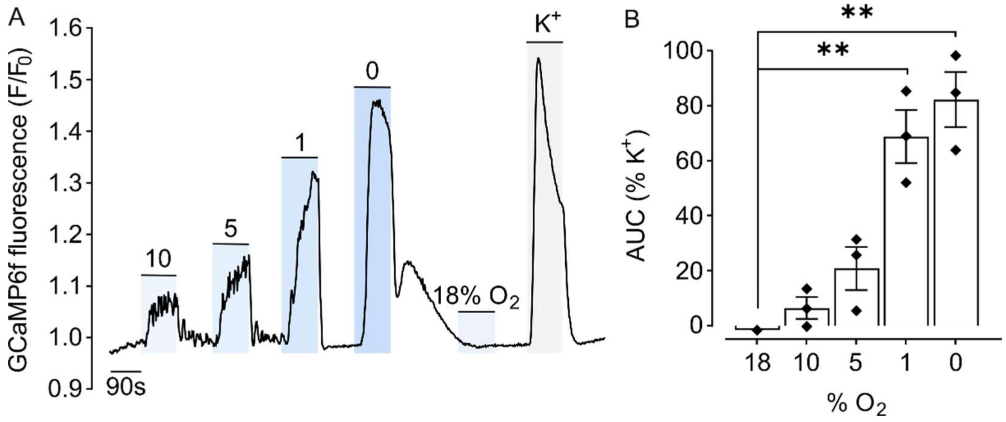

Supplementary Figure 2. Ca^{2+} imaging showing acute oxygen sensitivity in the carotid body from young adult mice expressing GCaMP6f in TH^+ cells (*Ai95$^{f/+}$;ThCre* or 'wild-type' mice). (**A**) Representative trace of F/F_0 GCaMP6f fluorescence in the K^+ responsive region of the carotid body exposed to various stimuli (denoted in blue/grey shaded areas): 10, 5, 1% O_2, anoxia, 45 mM K^+ or a sham (18% O_2) control. (**B**) Responses to hypoxia in the carotid body are quantified as the average area under curve, presented as % of the response to high K^+. Mean ± S.E.M. are plotted with individual values shown. Data were analysed by a one-way ANOVA, $P = 0.0005$, followed by Dunnett's multiple comparisons test, ** $P < 0.01$.

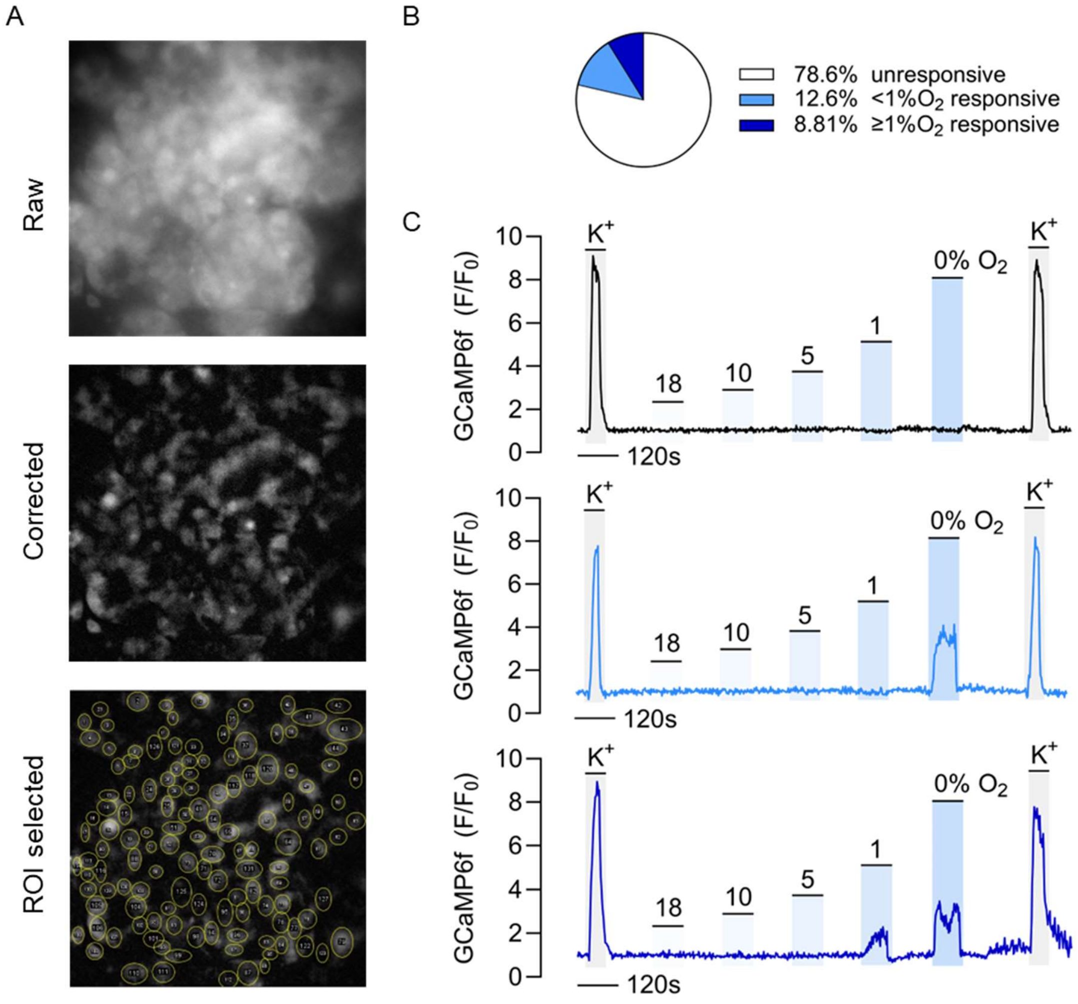

Supplementary Figure 3. High magnification Ca^{2+} imaging in wild-type adrenal medullae (AM) from young adult wild-type mice. (**A**) Representative raw, background corrected and regions of interest (ROI) annotated GCaMP6f fluorescence images in a wild-type AM slice following exposure to high K^+ viewed under x40 magnification. (**B**) Percentage of total K^+ responsive regions of interest that were: unresponsive to 0-10% O_2 ('unresponsive'); responsive to less than 1% O_2- ('<1%O_2 responsive') or responsive to at least 1% O_2 ('≥1%O_2 responsive'). Data summed from regions of interest imaged as per (A) from n = 4 adult wild-type mice and superfused with: 18%, 10%, 5%, 1% O_2, anoxia and 45 mM K^+. (**C**) Representative traces of unresponsive, <1%O_2 responsive and ≥1%O_2 responsive regions of interest showing F/F_0 fluorescence in the K^+ responsive regions of interest as the tissue is exposed to the indicated stimuli: 10-0% O_2, 45 mM K^+ or sham (18% O_2).

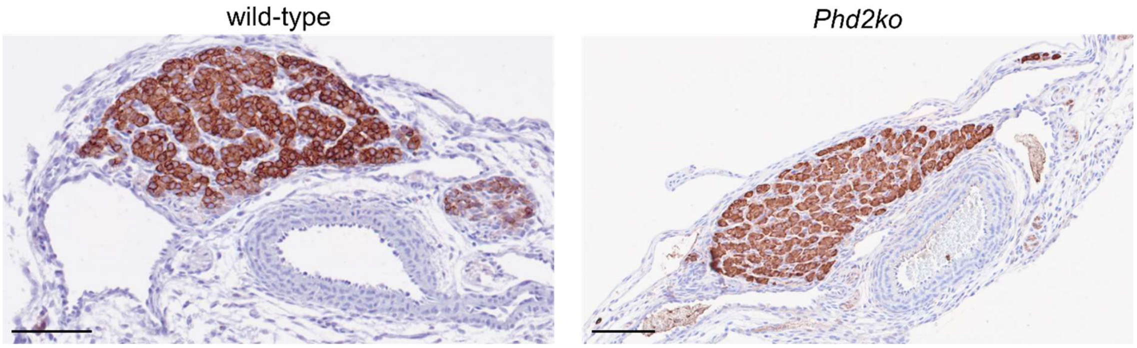

Supplementary Figure 4. Organ of Zuckerkandl (OZ) in neonatal wild-type (*Phd2$^{f/f}$*) and *Phd2ko* (*Phd2$^{f/f}$;ThCre*) mice. Representative images showing chromogranin A (CgA) immunohistochemistry (brown) in the OZ in neonatal (P0.5) wild-type and *Phd2ko* mice. Scale bars show 0.1 mm.

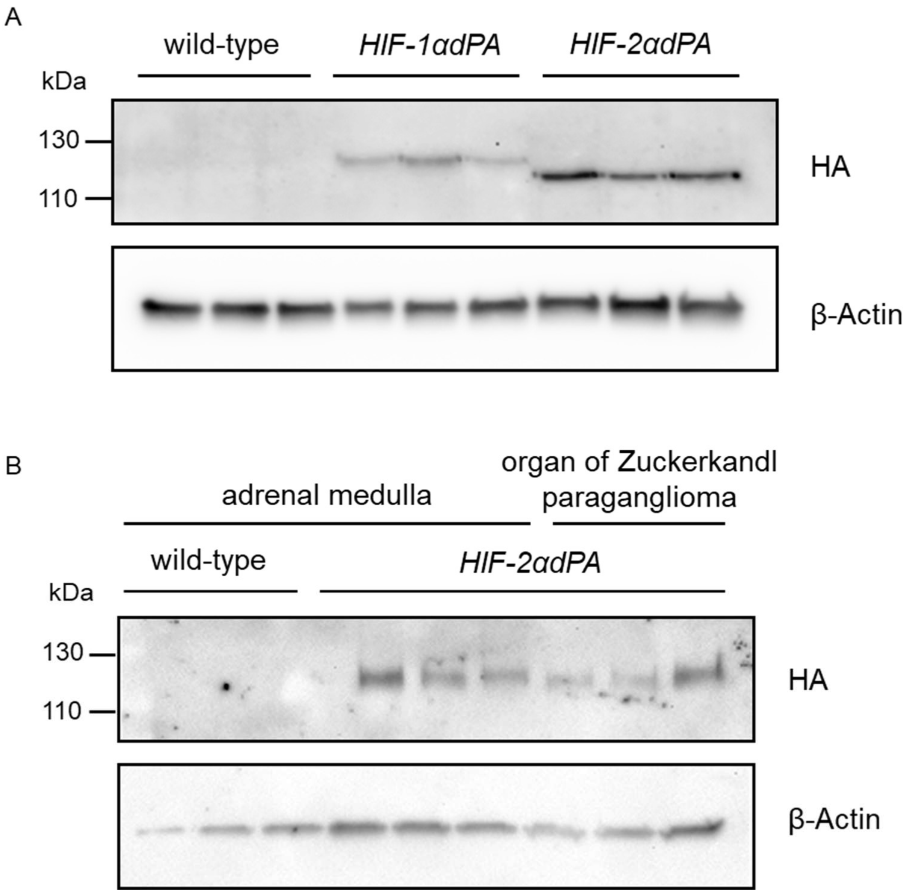

Supplementary Figure 5. HIF-1α and HIF-2α protein stabilisation in chromaffin tissues of *HIF-1αdPA* (*HIF-1αdPA$^{f/+}$;ThCre*) and *HIF-2αdPA* (*HIF-2αdPA$^{f/+}$;ThCre*) versus wild-type young adult mice. (A) Immunoblot for hemagglutinin (HA) or β-actin confirming HA-tagged *HIF-1αdPA* and *HIF-2αdPA* transgene expression in the adrenal medulla (AM) from *HIF-1αdPA* and *HIF-2αdPA*, but not wild-type, mice (n = 3 mice of each genotype, with one mouse per lane). **(B)** Immunoblot for hemagglutinin (HA) or β-actin confirming HA-tagged *HIF-2αdPA* transgene expression in the AM and organ of Zuckerkandl paraganglioma (OZ PGL) from *HIF-2αdPA*, but not wild-type, mice (n = 3 mice for AM and OZ, with one mouse per lane). The OZ PGL structure is not detected in adult wild-type and *HIF-1αdPA* mice).

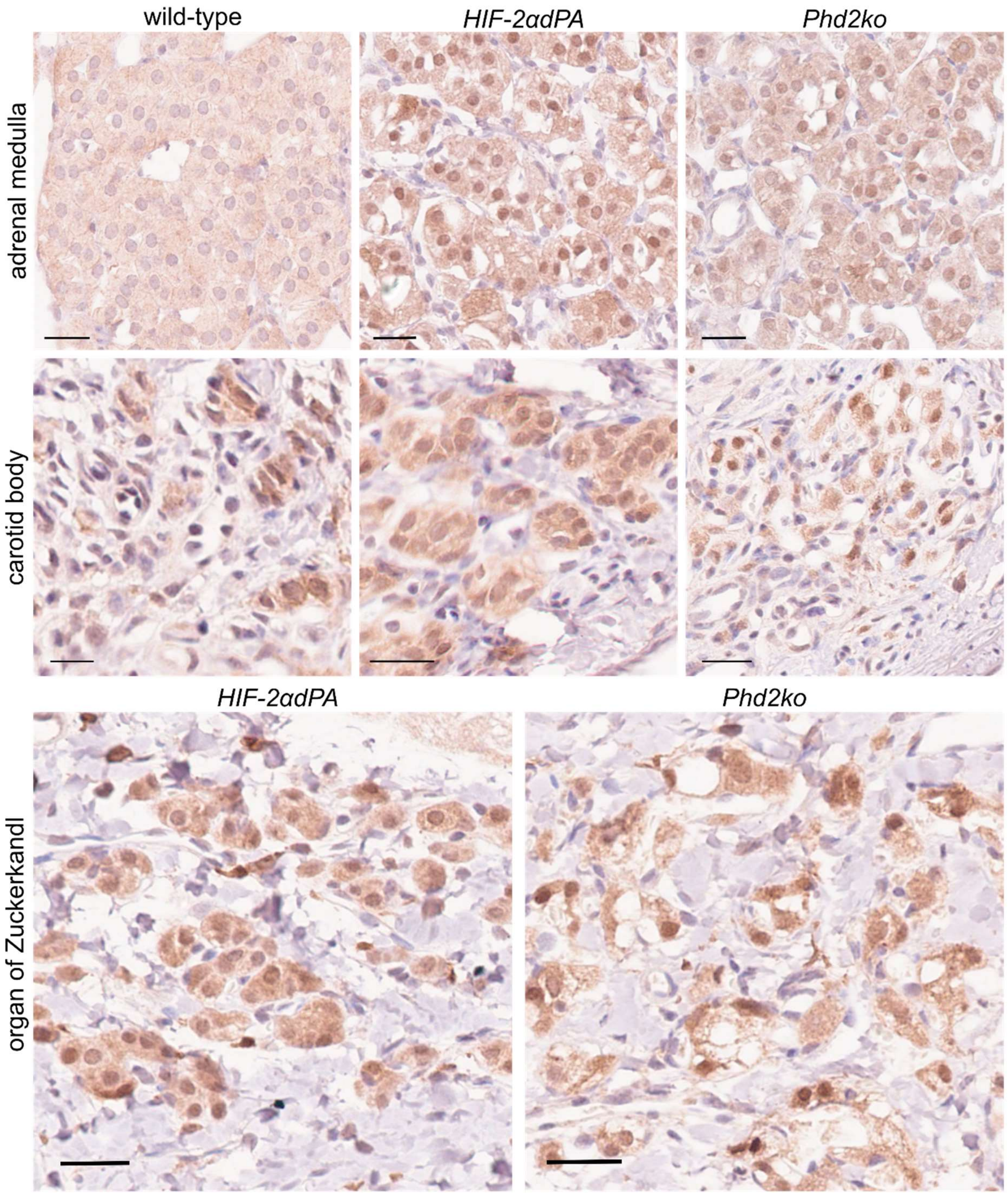

Supplementary Figure 6. HIF-2α protein stabilisation by immunohistochemistry in chromaffin tissues of young adult *HIF-2αdPA* (*HIF-2αdPA$^{f/+}$;ThCre*) and *Phd2ko* (*Phd2$^{f/f}$;ThCre*) mice. HIF-2α immunohistochemistry showing nuclear HIF-2α protein stabilisation in chromaffin cells from the adrenal medulla (top row), carotid body (second row) and organ of Zuckerkandl (bottom row) of *HIF-2αdPA* (middle) and *Phd2ko* (right), but not wild-type (left, where present), adult mice. Scale bars represent 0.025 mm.

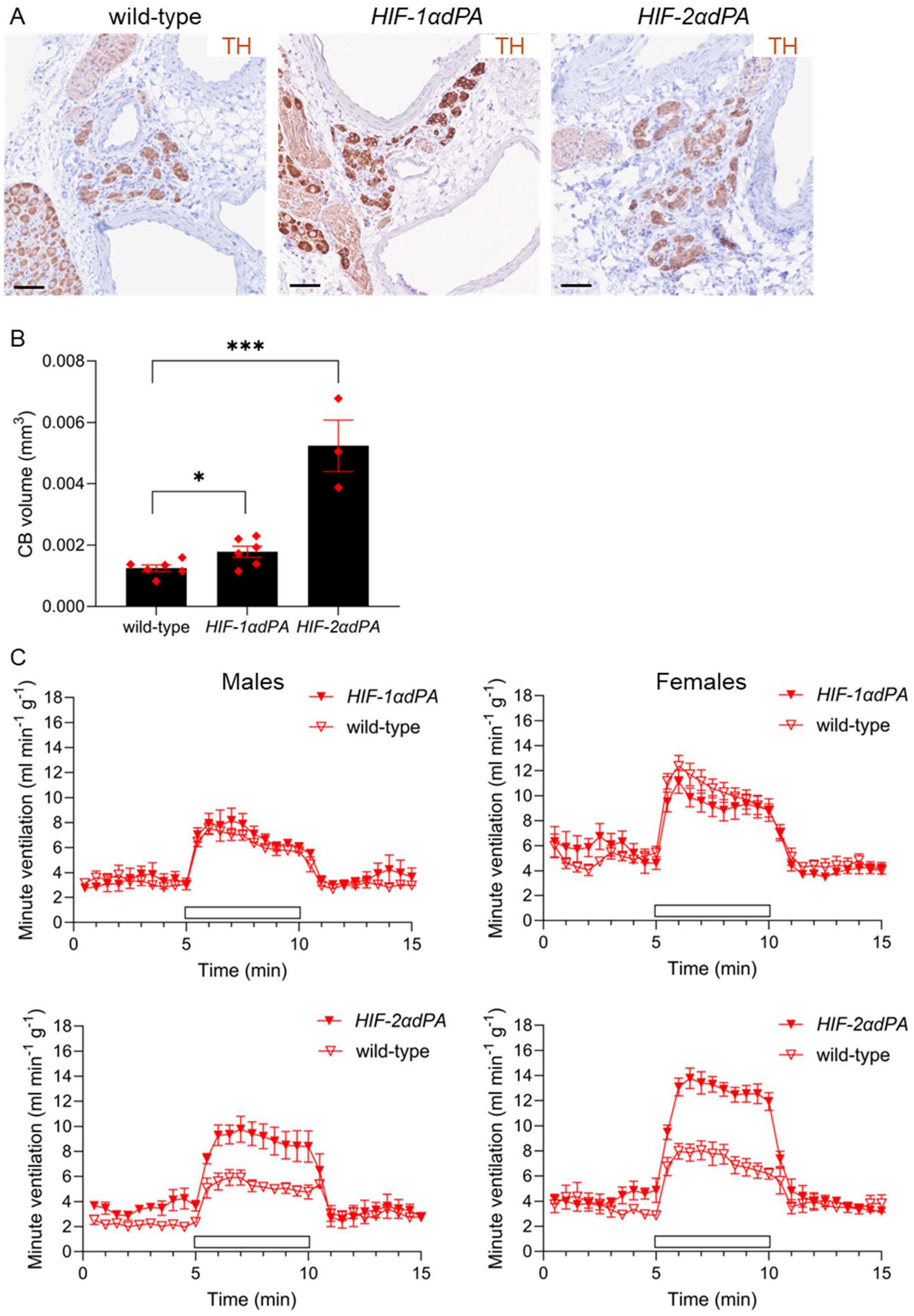

Supplementary Figure 7. Paraganglioma-like CBs and enhanced hypoxic ventilatory

responses in *HIF-1αdPA* (*HIF-1αdPA$^{f/+}$;ThCre*) and *HIF-2αdPA* (*HIF-2αdPA$^{f/+}$;ThCre*) compared to wild-type, young adult mice. (**A**) Tyrosine hydroxylase immunohistochemistry (TH)(brown) in CB sections from wild-type, *HIF-1αdPA* and *HIF-2αdPA* mice. Scale bars show 0.05 mm. (**B**) CB volume calculated from the area of TH^+ structures in sequential sections in adult wild-type, *HIF-1αdPA* and *HIF-2αdPA* mice. Bars show mean ± S.E.M. with individual values displayed. Data were analysed by an unpaired Student's *t*-test, * $P < 0.05$, *** $P < 0.001$. (**C**) Hypoxic ventilatory responses to 10% O_2/3% CO_2 (white box) in male (n = 5) and female (n = 6) *HIF-1αdPA* and *HIF-2αdPA* mice compared to wild-type mice. Acute response to hypoxia, quantified as the difference between minute ventilation in the first minute of stable hypoxia and the last minute of room air is: 8.7 ± 0.5 $ml \cdot min^{-1} \cdot g^{-1}$ in *HIF-2αdPA* versus 5.0 ± 0.6 $ml \cdot min^{-1} \cdot g^{-1}$ in wild-type females (mean ± S.E.M., unpaired Student's *t*-test $P = 0.0008$); 5.4 ± 0.5 $ml \cdot min^{-1} \cdot g^{-1}$ in *HIF-2αdPA* versus 3.6 ± 0.4 $ml \cdot min^{-1} \cdot g^{-1}$ in wild-type males ($P = 0.029$); 5.9 ± 0.9 $ml \cdot min^{-1} \cdot g^{-1}$ in *HIF-1αdPA* versus 6.7 ± 0.5 $ml \cdot min^{-1} \cdot g^{-1}$ in wild-type females ($P = 0.44$); 4.5 ± 1.0 $ml \cdot min^{-1} \cdot g^{-1}$ in *HIF-1αdPA* versus 4.4 ± 0.5 $ml \cdot min^{-1} \cdot g^{-1}$ in wild-type males ($P = 0.92$).

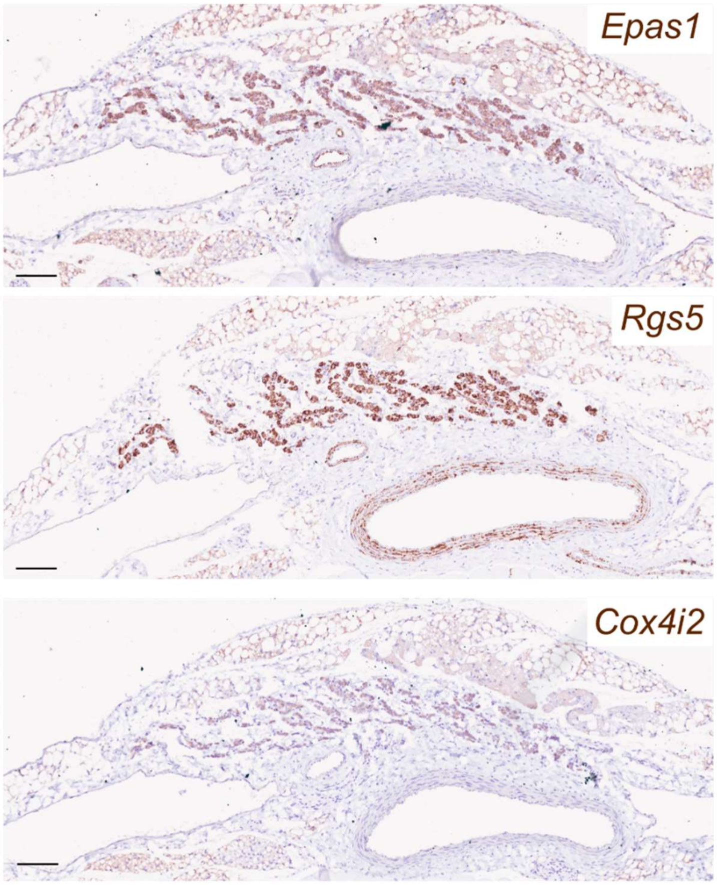

Supplemental Figure 8. Gene expression in abdominal organ of Zuckerkandl paraganglioma (OZ PGL) from young adult *HIF-2αdPA* (*HIF-2αdPA*$^{f/+}$*;ThCre*) mice. Representative images of *in situ* hybridisation for *Epas1, Rgs5* and *Cox4i2* mRNA (brown) in the OZ PGL from an adult *HIF-2αdPA* mouse. Scale bars are 0.1 mm.
