# Supplementary figures and images for: Hif-2α programs oxygen chemosensitivity in chromaffin cells
Source: J Clin Invest. 2024 Aug 6;134(18):e174661. doi: 10.1172/JCI174661 (PMC11405041; doi:10.1172/JCI174661)

Supplementary Figure 5A

HA

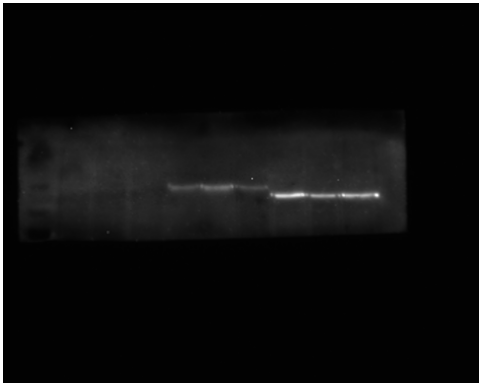

Bactin

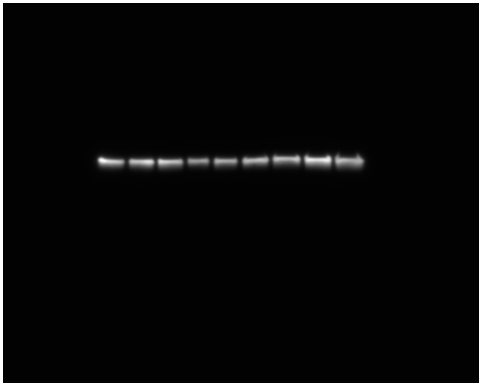

Supplementary Figure 5B

HA

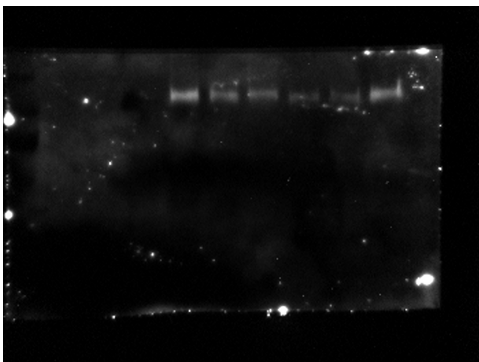

Bactin

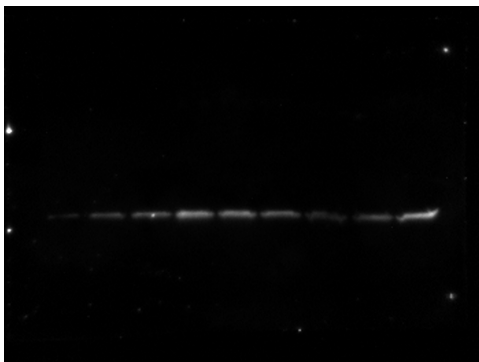

Supplement: Unedited blot and gel images [file jci-134-174661-s032.pdf]
